# Isolation and characterization of Hc-targeting chimeric heavy chain antibodies neutralizing botulinum neurotoxin type B

**DOI:** 10.3389/fimmu.2024.1380694

**Published:** 2024-04-30

**Authors:** Yujia Jiang, Rong Wang, Jiazheng Guo, Kexuan Cheng, Lei Chen, Xi Wang, Yating Li, Peng Du, Chen Gao, Jiansheng Lu, Yunzhou Yu, Zhixin Yang

**Affiliations:** Beijing Institute of Biotechnology, Beijing, China

**Keywords:** botulinum neurotoxin type B, Hc domain, phage display library, nanobody, heavy chain antibody, neutralizing antibody

## Abstract

**Background:**

Botulinum neurotoxin (BoNT) produced by *Clostridium botulinum* is one of the most potent known toxins. Moreover, BoNT is classified as one of the most important biological warfare agents that threatens the biosafety of the world. Currently, the approved treatment for botulism in humans is the use of polyvalent horse serum antitoxins. However, they are greatly limited because of insufficient supply and adverse reactions. Thus, treatment of human botulism requires the development of effective toxin-neutralizing antibodies. Considering their advantages, neutralizing nanobodies will play an increasing role as BoNTs therapeutics.

**Methods:**

Herein, neutralizing nanobodies binding to the heavy chain (Hc) domain of BoNT/B (BHc) were screened from a phage display library. Then, BoNT/B-specific clones were identified and fused with the human Fc fragment (hFc) to form chimeric heavy chain antibodies. Finally, the affinity, specificity, and neutralizing activity of antibodies against BoNT/B *in vivo* were evaluated.

**Results:**

The B5-hFc, B9-hFc and B12-hFc antibodies demonstrated high affinity for BHc in the nanomolar range. The three antibodies were proven to have potent neutralizing activity against BoNT/B *in vivo*.

**Conclusion:**

The results demonstrate that inhibiting toxin binding to the host receptor is an efficient strategy and the three antibodies could be used as candidates for the further development of drugs to prevent and treat botulism.

## 1 Introduction

Clostridial Neurotoxins are the most poisonous substances discovered so far, with lethal doses in the low ng/kg range (1, 2). Botulinum neurotoxins (BoNTs) are members of this family. Botulinum neurotoxins are very toxic to humans, leading to flaccid paralysis, a neuroparalytic disease with high mortality. According to the antigenicity of the toxin, BoNTs are divided into seven types (A-G) that are further subclassified as subtypes. Subsequently BoNT/H was discovered as a new chimeric serotype, comprising a hybrid-toxin of BoNT/A1 and BoNT/F5 (3–5). Serotypes A, B, E, and F are lethal and are responsible for most human botulinum cases (6). At present, most infant botulism is caused by botulinum neurotoxin type B (BoNT/B). A report covering data from 1978 to 2006 showed that botulinum type A and B accounted for 98.7% of all recorded cases of infant botulism worldwide (7), of which BoNT/B accounted for the highest proportion. The number of reported cases of type B botulism is still increasing (8). However, the progress in research on neutralizing antibodies against BoNT/B is not as extensive as that of BoNT/A. Therefore, strengthening research on the prevention and treatment of type B botulism is of great significance.

Botulinum neurotoxin is a comparatively inactive single chain polypeptide (150 kDa). After the toxins cross into the cytosol, they are proteolytically activated by a protease. Then, the disulfide bond is reduced, leading to the toxins being cleaved into a heavy chain (H chain, 100 kDa) and a light chain (L chain, 50 kDa). The H chain is composed of two functionally distinct regions: the C-terminal domain (Hc) and the N-terminal domain (HN) (9–11).The Hc domain of BoNT/B (BHc) plays a crucial role in botulism. Botulinum neurotoxins enter nerve cells via Hc binding to the surface of some residual nerve endings. When mice were immunized with fragments of three different functional regions of the toxin, several fragments located at the Hc domain could significantly protect mice, which provides a strong basis for the preparation of neutralizing antibodies (12).

Despite vaccination being an effective strategy to prevent botulism, other treatment methods against BoNTs in various pathological conditions are anticipated (6). To date, there is no small-molecule drug approved for either the prevention or post-intoxication treatment of botulism. Currently, polyvalent horse serum is the only available treatment for patients with botulism. The first FDA-approved antitoxin, BAT (Botulism Antitoxin Heptavalent, Equine) can neutralize all seven known BoNT serotypes (A–G). Neutralization of circulating BoNTs by BAT prevents further disease progression. However, BAT consists of equine-derived polyclonal IgG antibodies, which could cause various side effects and limits its effectiveness and reliability (11). An Investigational New Drug (IND) XOMA 3AB, which contains three human monoclonal antibodies, is effective in neutralizing BoNT/A (13). NTM-1632, which is an equimolar mixture of three human IgG monoclonal antibodies targeting BoNT/B, is under human clinical trial to assess its safety (14). However, monoclonal antibody drugs are generally expensive, require intravenous administration, and have limited shelf-lives. Therefore, alternative therapeutic antitoxin approaches that reduce costs and improve convenience remain an important goal.

At present, research on antibody therapy has a great prospect in clinical practice; however, antibodies are mainly limited by their large size and poor permeability in solid tissues. The single domain antibody, which lacks light chains and the heavy-chain lack CH1, referred to VHH or nanobody, was discovered in the serum of camelids (15, 16). As a novel and unique antigen-binding fragment, nanobodies have natural advantages that make them suitable to develop the next generation of biological drugs (17). Nanobodies are believed to be the smallest intact antigen binding fragment and recognize special epitopes involved in receptor recognition, reaching an extremely high-neutralization potency (18, 19). In addition, nanobodies typically exhibit a convex structure, which allows them to better identify and combine with other conformational epitopes that are not easily accessible. These conformational epitopes typically represent enzyme active sites and receptor binding domains (20, 21). The sequence consistency between nanobodies and the VH family III of human immunoglobulin exceeds 80% (22). Klarenbeek found that the sequence homology between camel immunoglobulin heavy-chain variable region family 3 and its human framework region counterpart was 95% (23). This means that the nanobodies will have a low immunogenic profile. Recently, some studies demonstrated that nanobodies can effectively inhibit the process of botulism in animal models (24–27). One drawback of nanobody is that due to their small size, they are quickly cleared by the kidneys. Therefore, many studies have fused several of nanobodies for expression or fused nanobodies with other proteins for expression to increase the molecular weight to a desired level (28–30). We speculated that the fusion of nanobodies with the Fc fragments of human antibodies to form chimeric heavy chain antibodies can potentially circumvent rapid renal clearance and have great neutralizing efficacy *in vivo*. This approach holds significant promise for enhancing the composition of advanced anti-BoNT therapeutic agents.

In the present study, three neutralizing nanobodies against BoNT/B, named B5, B9, and B12, were isolated from a phage display nanobody library and are specifically associated with the Hc domain of BoNT/B. After fusion with the human Fc fragment (hFc) to form chimeric heavy chain antibodies, B5-hFc, B9-hFc, and B12-hFc were proven to have potent neutralizing activity *in vivo*. The three antibodies have high potential for development as prophylactic and therapeutic antibodies against BoNT/B.

## 2 Materials and methods

### 2.1 Antigens, cell lines, and animals

Recombinant Hc proteins (AHc, BHc, EHc, and FHc) of botulinum toxin type A, B, E, and F were prepared in a previous laboratory study (31). Briefly, AHc, EHc, and FHc proteins expressed in *Escherichia coli* and the BHc protein expressed in yeast were purified by sequential chromatography. A 30 L pilot-scale purification of each Hc antigen was performed at the Pilot Production Base of our institute. The characteristics of the pilot-scale Hc antigens were determined, and qualified recombinant protein batches were stored at −80 °C for further studies. THc is the Hc domain of Tetanus toxin and 7f is the fiber protein of adenovirus serotype 7, which were prepared by our laboratory as controls (32, 33).

FreeStyle™ HEK293-F cells (Thermo Fisher Scientific, Waltham, MA, USA) were cultured in FreeStyle 293 expression medium (Thermo Fisher Scientific) at 37 °C and 5% CO_2_ to express the chimeric antibodies.

An adult male bimodal camel was sampled and provided by the Moshangke Camel Breeding Professional Cooperative in Fengnan District (Tangshan, China). We purchased female specific pathogen free (SPF) KM mice (18–20 g) from Beijing Spefo Biotechnology Co., Ltd. (Beijing, China) and raised them under SPF conditions in the Experimental Animal Center (Beijing, China). In the course of the experiment, we strictly abided by the 3R principle of animal experiments to ensure the basic rights of animal welfare.

### 2.2 Immunization of the camel

The camel was immunized with BHc prepared according to a previously described method (34). Briefly, purified BHc was mixed with Freud’s complete adjuvant in equal volumes. After full emulsification, the mixture was injected it into a healthy adult Bactrian camel at multiple points, with an interval of 14 days. After the first immunization, Freud’s incomplete adjuvant was used in the rest of the immunizations. After four immunizations, camel serum was collected. An enzyme-linked immunosorbent assay (ELISA) method was used to detect the specific antibody titer against BHc in the camel serum. The serum before immunization was used as the negative control. After determining that a high antibody titer had been obtained, a fifth immunization was carried out. One week after the last immunization, 120 mL camel blood sample was collected to isolate the peripheral blood B lymphocytes (PBMCs).

### 2.3 Construction of the phage display nanobody library

The total RNA from PBMCs of the immunized camel was extracted. The cDNA was used as a template to amplify the nanobody fragments using an RT-PCR reverse transcription kit (Invitrogen Superscript III; Waltham, MA, USA). Specific primers were designed to obtain the nanobody fragments through two rounds of nested PCR. The obtained nanobody fragments were digested with Nco I/Not I and cloned into the ScFv-NEN expression phage vector (32). The recombinant phage was introduced into newly prepared *E. coli* TG1 competent cells via electroporation. The transformed cells were titrated on a 2-YT Agar plate, and some single colonies were selected for sequencing to detect the insertion of nanobody fragments. Cells containing a phage display nanobody library with diverse sequences were collected, packaged, and stored at −80 °C.

### 2.4 Biopanning and isolation of the BHc-binding nanobodies

Bacteria from the phage display nanobody library were added into 2-YT-GA medium (with 100 μg/mL ampicillin and 2% glucose) and incubated. M13K07 max helper phages were added to the bacteria to display the nanobody libraries. The bacteria were centrifuged, the pellet was resuspended in 2-YT-KAA medium (with 100 μg/mL ampicillin, 100 μg/mL kanamycin, and 0.001% arabinose), and cultured overnight in a culture shaker at 220 rpm at 28°C. The phage antibody library was then purified. After the bacteria were centrifuged, the phages in the supernatant were precipitated with 20% polyethylene glycol (PEG) 6000 in 2.5 M NaCl, and the precipitates were washed using phosphate-buffered saline (PBS). The newly prepared phage antibody library was infected into TG1 bacteria (OD ≈ 0.4) prepared on the same day, and then cultured at 37 °C for 30 min at 150 rpm. The mixture was spread on a 2-YT-GA solid plate and cultured overnight at 37 °C to calculate the titer of the phage antibody library. The BHc protein was incubated overnight in 0.05 M carbonate buffer (pH = 9.6) at 4 °C. Immune tubes were coated with 20 μg, 10 μg, and 5 μg BHc antigens, respectively, for three rounds of biopanning. After sealing the immune tube with a blocking solution (PBS with 100 μg/mL bovine serum albumin) at 21 °C for 2 h, the phage library solution was added to the immune tubes and incubated at 21 °C for 2 h. Thereafter, the tubes were rinsed with PBS or PBST (PBS with 0. 5% Tween-20). Nanobodies in the immune tube were eluted using 0.1M Glycine-HCl (pH = 2.2) and then infected into TG1 cells at 37°C for 30 min. Part of the infected cells were used for enrichment statistics, and the rest was used for the next round of biopanning. The amount of phage used in each panning represented the input of the round. The number of clones after each panning represented the output of the round. The ratio of the input/output ratio of each round to the input/output ratio of the previous round was used as the enrichment factor for each round.

After three rounds of screening, the BHc protein was used as the antigen for Phage Enzyme-Linked Immunosorbent Assay (Phage-ELISA). The positive clones were screened, sequenced, and analyzed to obtain several sequences of nanobodies that specifically targeted BHc.

### 2.5 Construction, expression, and purification of the heavy chain antibodies

The positive clones were screened as templates, primers were designed to amplify the gene fragment of the variable domain of heavy chain of the heavy chain antibody (VHH), which was then inserted into an expression vector containing a human-derived antibody Fc fragment. With the help of FectoPRO transfection reagent, the recombinant VHH-hFc fusion protein expression plasmids were transfected into HEK-293 cells. The cell activity was monitored daily after 72 h. When the cell activity decreased from 95–100% to 80–85%, the cell supernatant was centrifuged at 8000 g for 10 min, and the supernatant was harvested. The heavy chain antibodies were purified using the AKTA Pure purification system in two steps: HiTrap™ MabSelect Xtra and HiTrap™ Desalting. Then, the molecular weight of the heavy chain antibodies was determined using sodium dodecyl sulfate-polyacrylamide gel electrophoresis (SDS-PAGE).

### 2.6 Cross-reaction and binding activity of the heavy chain antibodies

The 96-well plates were coated with BHc or other antigens whose concentration was adjusted to 2 μg/mL with 50 mM carbonate coating buffer, added at 100 μL/well to the wells, and incubated overnight at 4 °C. After washing six times with PBST, the plates were blocked with PBS containing 3% skim milk for 2 h at 37°C and then the blocking solution was discarded. After washing with PBST six times, the antibodies to be tested (in PBS containing 3% skim milk) were added at 100 μL/well and incubated at 37°C for 1.5 h. The primary antibody solution was discarded, the wells were washed six times with PBST, and 100 μL/well of horseradish peroxidase-labeled goat anti-human IgG diluted with 3% skim milk (1:4000) was added and incubated for 45 min at 37 °C. The secondary antibody solution was discarded, the wells were washed six times with PBST, peroxidase substrate chromogenic solution was added at 50 μL/well, and the color was allowed to develop for 18 min in the dark. The reaction was stopped using 2 M H_2_SO_4_ at 50 µL/well and the absorbance was measured at 492/630 nm using a microplate reader (Molecular Devices, Sunnyvale, CA, USA).

The steps of binding activity assay were described above in detail except that the antibodies were diluted to 12.5 μg/mL and then two-fold diluted 18 times. The relative binding activity of the subject antibodies to BHc was determined as the effective concentration of the antibodies (EC_50_) and curve fitting was performed using GraphPad Prism software 8 (version 8.0.2.263, GraphPad Inc., San Diego, CA, USA).

### 2.7 KD analysis and cross-competitive binding assay

The binding of B5-hFc, B9-hFc, B12-hFc, B14-hFc, B20-hFc antibodies to BHc antigen was separately measured using ForteBIO^®^ Octet QKe System (Pall ForteBio Corporation), a biolayer interferometer based on biological layer interferometry (BLI). The antibodies diluted to 200 nM with HBS-EP buffer were immobilized on the Anti-hIgG Fc Capture (AHC) biosensors during a 300 s loading step. Following a 60 s baseline step in HBS-EP buffer, the antibody-coated sensors were individually dipped into wells containing BHc antigen at the seven titrated concentrations (ranging from 500 to 7.8125 nM with two-fold dilutions) for a 90 s-130 s Association step. Subsequently, the Dissociation step was performed in HBS-EP buffer for 40 s-80 s. The values of KD, kon and kdis were evaluated using a 1:1 binding model and the ForteBio Data Analysis Software 7.0 (Pall ForteBio Corporation).

A competitive binding experiment was also conducted to determine whether the binding regions of the five antibodies on the BHc antigen surface were the same. When B5-hFc was detected, B5-hFc diluted to 200 nM with HBS-EP buffer was immobilized on the AHC biosensors during a loading step. Following a 60 s baseline step in HBS-EP buffer, an Association step with BHc antigen diluted to 400 nM in HBS-EP buffer was carried out before the Re-association step. Subsequently, a Re-association step was conducted in the sample plate with 200 nM B5-hFc, B9-hFc, B12-hFc, B14-hFc, and B20-hFc, along with 200 nM T23-hFc or HBS‐EP buffer as a control. T23-hFc was a heavy chain antibody specific to Hc domain of tetanus toxin (TeNT). The reverse experiment with B9-hFc, B12-hFc, B14-hFc, and B20-hFc captured on AHC biosensors was conducted in a similar manner. The results were analyzed using a 1:1 binding model and the ForteBio Data Analysis Software 7.0 (Pall ForteBio Corporation).

### 2.8 Neutralizing activity *in vivo*

The neutralizing activity of the antibodies was evaluated using mouse experiments. During the initial screening for neutralizing antibodies, the 24 purified antibodies were diluted in a dilution buffer (50 mM KH_2_PO_4_, 50 mM Na_2_HPO_4_, 1 M NaCl, 1% gelatin, pH 6.5) to three different doses (10 μg, 5 μg, and 1 μg). These diluted antibodies were then premixed with a standardized 20 × LD_50_ BoNT/B solution, resulting in a final volume of 500 μL per mixture. Subsequently, the mixtures were incubated at 37°C for 30 minutes. The negative control group was the mixture of BoNT/B with dilution buffer. Moreover, an irrelevant antibody, T23-hFc also served as a negative control. SPF female KM mice (18–20g) were randomly divided into groups (four mice/group) and injected intraperitoneally (i.p.) with 500 μL of the mixture per mouse. The health of the mice was continuously monitored and deaths were recorded over 7 days.

The antibodies with good neutralization activity were further tested in a dose-dependent manner to calculate the neutralization titer. The antibodies were diluted to six different doses (2 µg, 1 µg, 0.5 µg, 0.25 µg, 0.125 µg, and 0.0625 µg) and each was pre-incubated individually with 20 × LD_50_ BoNT/B. Each mixture has a total volume of 500 μL. The subsequent mouse experiments were carried out as described above. We calculated the 50% effective concentration (ED_50_) value, expressed as IU/mg. An standard F(ab’)_2_ antitoxin called BAT-B from the serum of highly immunized horses against BoNT/B served as a positive control, which was prepared in our previous study (35).

### 2.9 Preventive and therapeutic effects of antibodies in a mouse model

Female SPF KM mice (18–20 g) were randomly divided into groups (n = 4 per group). To evaluate the preventive effect, the antibodies (0.025 or 0.125 mg/kg) were administered through tail vein of mice. After 1, 2, or 3 days of injection of the antibodies, different doses of BoNT/B (20 or 100 × LD_50_) were injected i.p. into the experimental mice. The health of the mice was continuously monitored and deaths were recorded over 7 days. The positive control comprised BAT-B, an F(ab’)2 antitoxin from the serum of highly immunized horses against BoNT/B. The negative control comprised T23-hFc.

To evaluate the therapeutic effect, different doses of BoNT/B (5 or 20 × LD_50_) were injected i.p. into female KM mice. After 0.5, 1, 2, or 3 h of exposure to BoNT/B, the mice were administered with the antibodies (0.025 or 0.125 mg/kg) via their tail vein. The positive and negative controls comprised BAT-B and T23-hFc, respectively. The health of the mice was continuously monitored and deaths were recorded over 7 days.

### 2.10 Statistical analysis

The data from the prophylactic and therapeutic efficacy experiments were analyzed using GraphPad Prism software. The Log-rank test was used to evaluate the significance of the difference in protection compared with the control group. Statistical significance was accepted when the p value of the difference was < 0.05.

## 3 Results

### 3.1 Construction and screening of the phage display nanobody library

**Figure 1:**
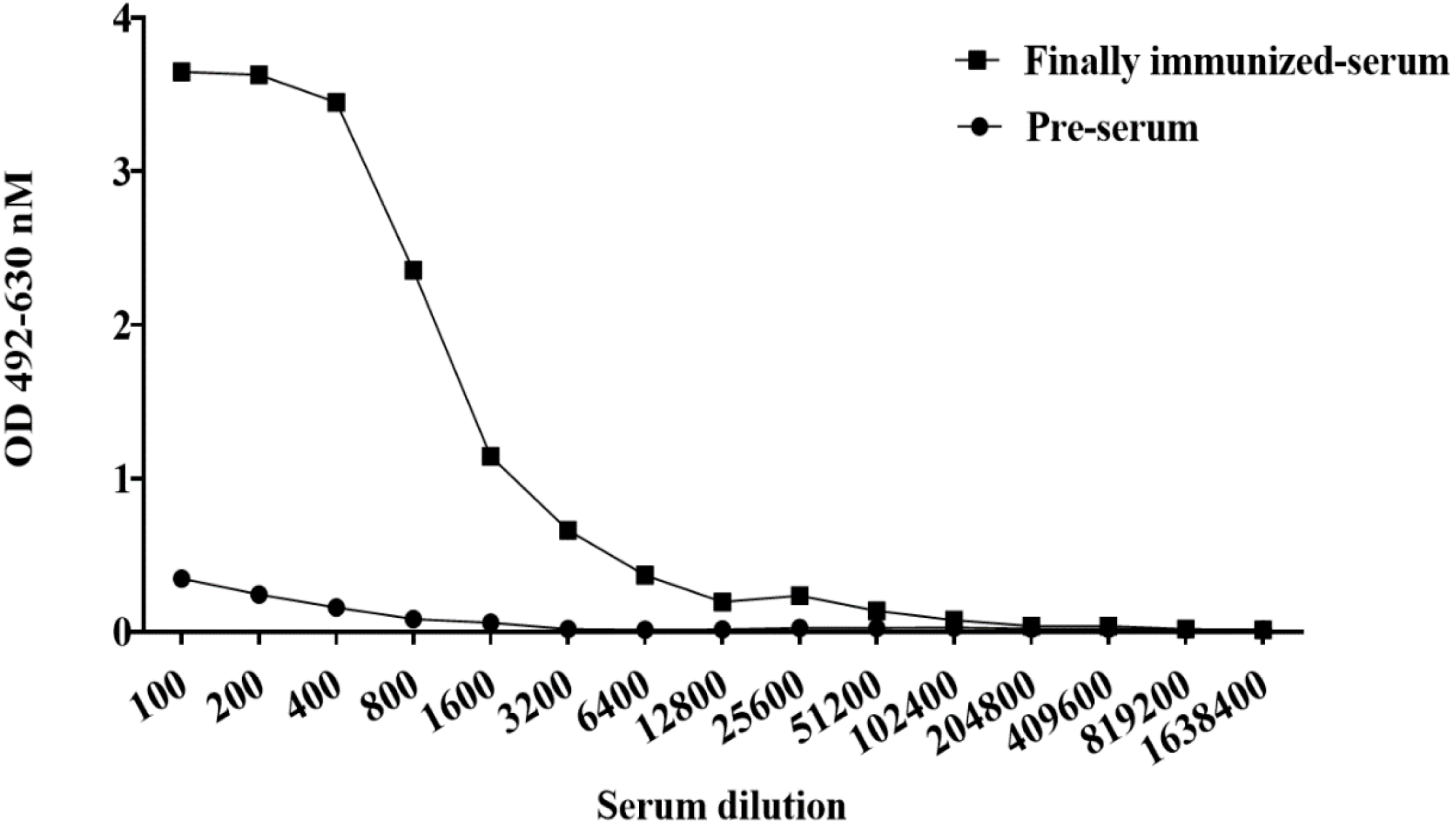
ELISA was used to detect the binding activity against BHc antigen produced in camel serum. When the ratio of (OD value of positive serum - OD value of blank) to (OD value of negative control - OD value of blank) was more than 2, the well was usually defined as positive and the maximum serum dilution in the positive wells represented the antibody titer.

To obtain neutralizing antibodies with high specificity, diversity and affinity, purified BHc was used to immunize Bactrian camels. Compared with the pre-immunization serum, the serum after five immunizations had a titer of approximately 1:51200 (**Figure 1**). The results showed that the antibody titers increased significantly after immunization and showed effective and specific serological activity toward BHc. 120 mL camel peripheral blood was collected within one week after booster immunization. Total PBMCs were isolated to extract total RNA. Amplified nanobodies encoding fragments were ligated into an expression vector and electrotransformed to construct *E. coli* libraries containing the nanobody fragments. Finally, we constructed a phage display nanobody library against BoNT/B. The capacity of the bacterial library exceeded 4 × 10^8^ colony forming units (CFU).

Using BHc as the antigen, three rounds of panning were carried out. The screening conditions of each round gradually became stricter, the amount of antigens used in the screening library decreased, and the washing conditions increased; however, the corresponding input-output ratios increased. After three rounds of screening, the enrichment factor reached 1.6 × 10^4^, which showed that the nanobodies were effectively enriched (**Table 1**).

**Table 1:** Enrichment and panning of the phage display nanobody library.

Panning Round	Input^a^ (cfu)	Output^b^ (cfu)	Input/Output Ratio^c^	Enrichment Factor^d^
1	5 × 10^11^	8.4 × 10^5^	1.68 × 10^-6^	
2	1.2 × 10^11^	2 × 10^8^	1.7 × 10^-3^	1.0 × 10^3^
3	1.2 × 10^11^	3.24 × 10^9^	2.7 × 10^-2^	1.6 × 10^4^

a.The amount of phage added in each round of panning.

b.The amount of phage produced in each round of panning.

c.The input-output ratio of phages in each round of panning.

d. Ratio of the input-output ratio of two adjacent panning.

**Figure 2:**
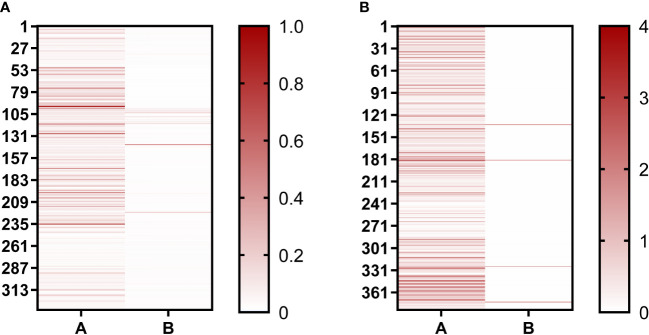
Positive clones were identified using Phage-ELISA. **(A)** Identification of single clones after the second round of screening; **(B)** Identification of single clones after the third round of screening. “A” represents clones binding to the BHc antigen; “B” represents clones binding to the BSA antigen. The darker the red color, the higher the OD492nm/630nm value.

In our study, phage-ELISA was used to select the nanobody clones that can only bind to BHc after the second and third round of panning, with BSA as antigen control. A total of 336 clones were selected after the second round of panning, of which 119 clones were positive (positive rate = 35%) (**Figure 2**). After the third round of panning, 376 clones were selected and 346 were positive (positive rate = 92%). This proved that the nanobodies were well enriched after screening. After sequencing, the sequences of the nanobodies were analyzed using IMGT/V-QUEST web page. The diversity of complementarity determining region (CDR3) sequences enable VHHs to be specific, thus the nanobodies would show different immune effects. According to the difference sequences of CDR3, finally, 72 sequences were obtained and named B1–B72. The sequences from B1 to B24 were repeated more than twice in the third round of panning, and these sequences were selected for further study.

### 3.2 Expression and purification of the heavy chain antibodies

**Figure 3:**
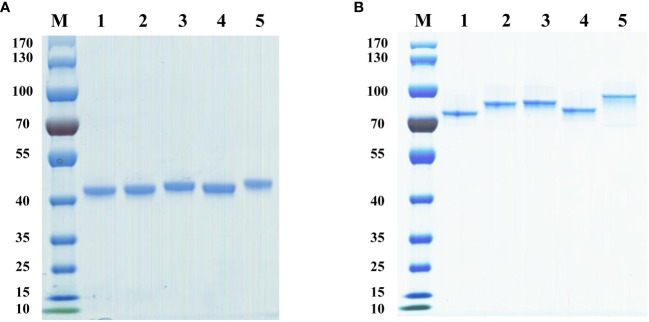
Analysis of diverse purified heavy chain antibodies using SDS-PAGE. **(A)** SDS-PAGE under reducing conditions. Lane 1, B5-hFc; lane 2, B9-hFc; lane 3, B12-hFc, lane 4, B14-hFc, lane 5, B20-hFc; M, protein markers. **(B)** SDS-PAGE under non-reducing conditions. Lane 1, B5-hFc; lane 2, B9-hFc; lane 3, B12-hFc, lane 4, B14-hFc, lane 5, B20-hFc; M, protein markers.

The expression plasmids of chimeric heavy chain antibodies were constructed using the positive clonal cultured supernatant of B1 to B24, which had been well enriched, as the template. Then, the plasmids were expressed in FreeStyle HEK293-F cells. The antibodies were purified using the AKTA Pure purification system, and the molecular weight of the heavy chain antibodies were determined using SDS-PAGE. The results showed that the molecular weight of these heavy chain antibodies were 40 kDa under reducing conditions (**Figure 3A**) and 80 kDa under non-reducing conditions (**Figure 3B**), respectively, which corresponded to the theoretical molecular weight of the heavy chain antibody.

### 3.3 Preliminary evaluation of the antibodies with potency neutralizing activity *in vivo*

Mice were used to detect the neutralizing activity of the antibodies. We pre-incubated 24 purified antibodies with BoNT/B before injection to evaluate the neutralization activity of the anti-BHc antibodies. The negative antibody control comprised BoNT/B mixed with T23-hFc. At a dose of 10 μg, 12 antibodies could completely protect mice against a 20 × LD_50_ challenge of BoNT/B. However, when the antibody dose was 1 μg per mouse, only B5-hFc, B9-hFc, B12-hFc, B14-hFc, and B20-hFc could completely protect the mice from exposure to 20 × LD_50_ of BoNT/B. The results showed the great potential neutralizing potency of these antibodies against BoNT/B (**Table 2**).

**Table 2:** Neutralizing activity of the antibodies against BoNT/B.

Group	Number of survivors^a^/Total mice per group^b^			Group	Number of survivors^a^/Total mice per group^b^		
	10 μg^c^	5 μg^c^	1 μg^c^		10 μg^c^	5 μg^c^	1 μg^c^
B1-hFc	1/4	0/4	0/4	B14-hFc	4/4	4/4	4/4
B2-hFc	4/4	4/4	0/4	B15-hFc	2/4	1/4	0/4
B3-hFc	1/4	1/4	0/4	B16-hFc	4/4	4/4	1/4
B4-hFc	1/4	0/4	0/4	B17-hFc	1/4	1/4	0/4
B5-hFc	4/4	4/4	4/4	B18-hFc	4/4	2/4	0/4
B6-hFc	4/4	4/4	0/4	B19-hFc	1/4	0/4	0/4
B7-hFc	0/4	0/4	0/4	B20-hFc	4/4	4/4	4/4
B8-hFc	2/4	1/4	0/4	B21-hFc	1/4	0/4	0/4
B9-hFc	4/4	4/4	4/4	B22-hFc	4/4	4/4	1/4
B10-hFc	4/4	4/4	0/4	B23-hFc	4/4	4/4	1/4
B11-hFc	0/4	0/4	0/4	B24-hFc	1/4	0/4	0/4
B12-hFc	4/4	4/4	4/4	PBS	0/4	0/4	0/4
B13-hFc	1/4	0/4	0/4	T23-hFc	0/4	0/4	0/4

a. 20 × LD_50_ BoNT/B was pre-incubated with 10 μg, 5 μg, or 1 μg of antibody, separately. The mixed solution comprised a total of 500 μL. The data in the table show the number of surviving mice at 7 days after intraperitoneal injection of 500 μL of the mixture.

b. There were four mice per group.

c. Different doses of antibody mixed with 20 × LD_50_ BoNT/B were injected into each mouse.

### 3.4 Binding activity and cross-reactivity of the antibodies

**Figure 4:**
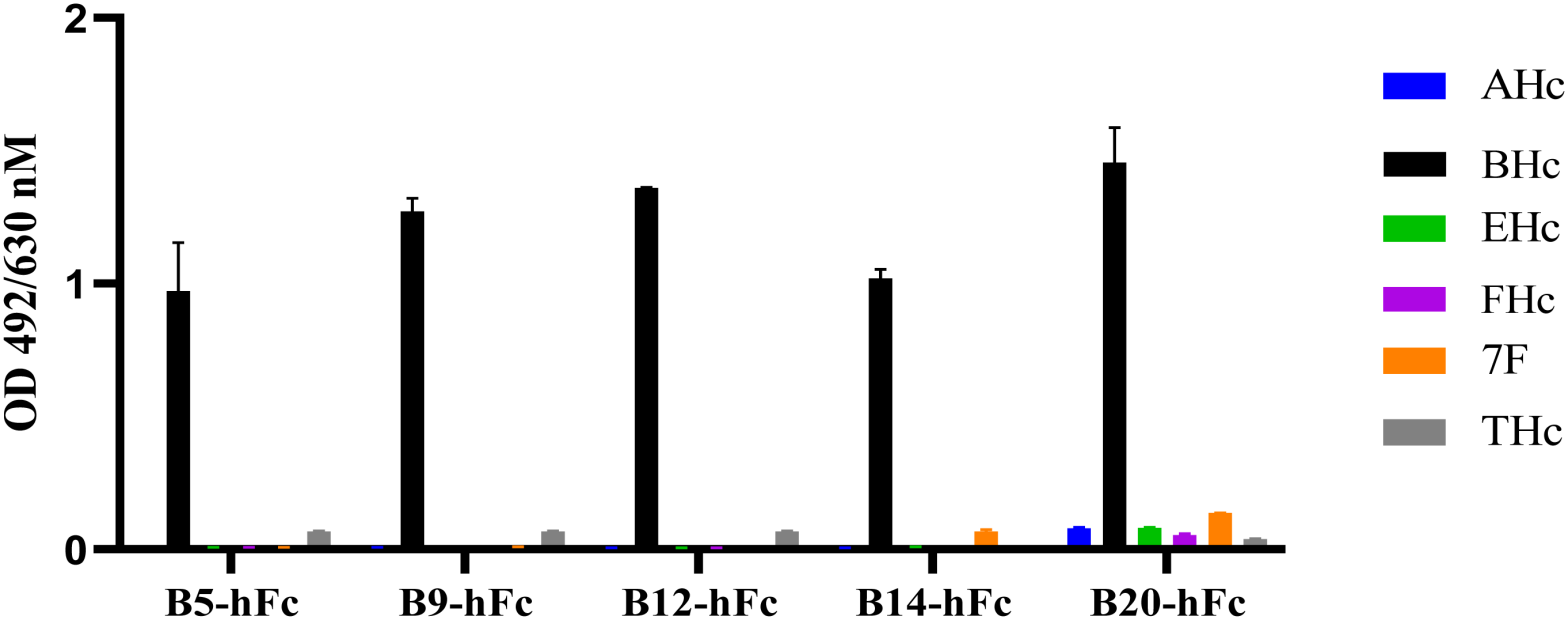
Specificity of the B5-hFc, B9-hFc, B12-hFc, B14-hFc and B20-hFc. The binding between the antibodies and different antigens. The concentration of antigens and antibodies were all 2 μg/mL.

Binding activity is also one of the important indicators of antibody activity. In order to further verify the biological activity of heavy chain antibodies, the binding activity of antibodies were determined using ELISA. All antibodies bound to BHc in a concentration-dependent manner. The results indicated that the EC_50_ values of the five antibodies (B5-hFc, B9-hFc, B12-hFc, B14-hFc and B20-hFc), which demonstrated significant neutralizing activity, ranged between 0.1 and 3 nM. In contrast, the binding activity of the remaining antibodies was generally weak. Specifically, for B11-hFc, B13-hFc and B15-hFc, it is hypothesized that they may bind to non-neutralizing epitopes (**Supplementary Figure 1**). The antigens tested comprised AHc, BHc, EHc, FHc, THc, and the fiber protein of adenovirus serotype 7. Under the similar detection conditions, the specificity of binding between the antibody and BHc was analyzed by ELISA. The results indicated that B5-hFc, B9-hFc, B12-hFc, B14-hFc, and B20-hFc specifically bound to BHc, but did not cross-react with the other antigens (**Figure 4**).

### 3.5 Affinity and competitive binding activity between different antibodies

In order to accurately analyze the affinity of heavy chain antibodies, we conducted a BLI assay to determine the binding affinities of the five antibodies to the BHc antigen. The results showed that all antibodies exhibited a gradual decrease during the dissociation phase, indicating significant binding kinetics of antibodies to BHc (**Supplementary Figure 2**). According to the **Table 3**, the results suggested that the antibodies have affinities to BHc with KD values ranging from 4 nM to 13 nM. In addition, the activity of B9-hFc binding to BHc was strongest.

**Table 3:** The binding kinetics of the antibodies against BoNT/B.

	Mean			
Antibody	kon(10^4^ Ms^-1^)^a^	kdis(10^-4^ s^-1^)^b^	KD(10^-9^ M)^c^	>χ^2^
B5-hFc	5.86	4.68	7.99	0.18
B9-hFc	7.89	3.56	4.51	0.29
B12-hFc	7.47	9.08	12.20	0.16
B14-hFc	7.40	6.07	8.20	0.44
B20-hFc	7.83	6.47	8.26	0.56

a. kon means association constant.

b. kdis means dissociation constant.

c. KD which is the ratio of kdis to kon means equilibrium dissociation constant.

**Figure 5:**
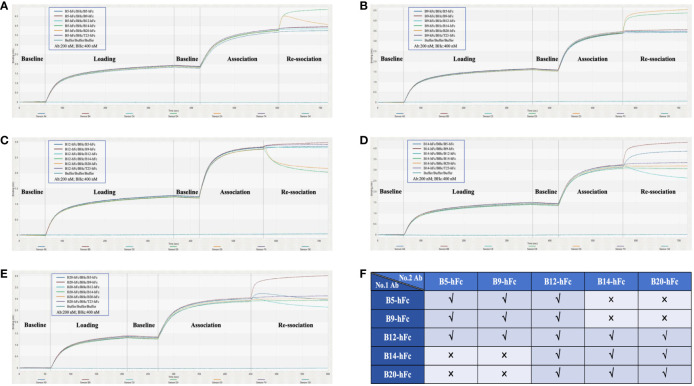
Competitive binding analysis of five antibodies with BHc using BLI. This figure illustrates the dynamic kinetic process of competitive binding among various antibodies to BHc. Antibodies were immobilized on the biosensor for determination. The antibodies included are B5-hFc **(A)**, B9-hFc **(B)**, B12-hFc **(C)**, B14-hFc **(D)**, and B20-hFc **(E)**. As detailed in the legend, stages of the assay were demarcated by slashes “/”, specifically denoting the “Loading”, “Association”, and “Re-association” phases. **(F)** delineates the competitive interaction amongst the five antibodies and BHc. A “√” signifies that the respective second antibody competitively binds to BHc in the presence of a first antibody, whereas an “x” denotes the absence of competitive binding between the first and second antibodies.

We also conducted competitive binding assays to determine whether there was a completion between any two of the five antibodies to bind BHc. Even when the binding between B9-hFc and BHc reached saturation, B14-hFc and B20-hFc continued to bind to BHc, leading to an increase in signal (**Figure 5**). When the latter two antibodies were immobilized on biosensors, the results were consistent. These results indicated that B9-hFc did not compete with B14-hFc and B20-hFc for binding to BHc. Similarly, B5-hFc and B14-hFc did not exhibit competitive binding to BHc. Additionally, there were instances where the signal initially increased and then decreased during the disassociation step (**Figures 5A, E**). The results also suggested that B5-hFc did not compete with B20-hFc for binding to BHc. The competitive binding of BHc between five antibodies was summarized in **Figure 5F**.

### 3.6 Neutralizing activity *in vivo*

**Figure 6:**
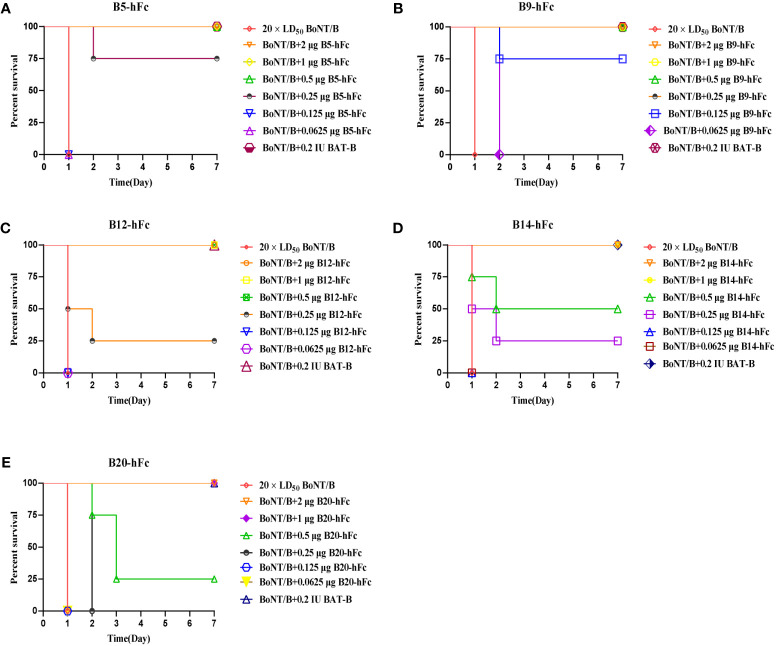
Evaluation of antibody neutralization efficiency. Each mouse was injected 500 mL mixture, which means that the five antibodies at descending dosages of 2 μg, 1 μg, 0.5 μg, 0.25 μg, 0.125 μμg, and 0.0625 μg were seperately mixed with 20 × LD50 BoNT/B. The five antibodies are B5-hFc **(A)**, B9-hFc **(B)**, B12-hFc **(C)**, B14-hFc **(D)**, and B20-hFc **(E)**. The figure presents the survival rate of mice against BoNT/B in different concentrations of antibodies.

We further evaluated the neutralizing activity of the five antibodies against BoNT/B in KM mice. The antibodies were diluted to concentrations of 2 µg, 1 µg, 0.5 µg, 0.25 µg, 0.125 µg, and 0.0625 µg, and each was individually pre-incubated with BoNT/B at a dose of 20×LD_50_. After administering 500 μL of these mixtures via intraperitoneal injection, we observed the survival rates of mice in groups of four. BoNT/B mixed with diluent was used as a negative control (**Figure 6**). A standard antitoxin for BoNT/B was included as a positive control. The efficacy of the antibodies was compared to the reference standard antitoxin of BoNT/B. The neutralizing ability of anti-BoNT products are estimated in international units (IU), and 1 IU of antitoxin protects mice from 10000 × LD_50_ BoNT/B (36). In our study, the antitoxin potency was calculated as IU/mg, 1 IU/mg means 1 mg antibody neutralize 10000 × LD_50_ BoNT/B (37). The results showed that 0.66 µg of B20-hFc could provide 50% protection against 20 × LD_50_ BoNT/B, the theoretically neutralization capacity was approximately 30300 × LD_50_/mg, which was the equivalent of 3 IU/mg; 0.5 µg of B14-hFc could provide 50% protection against 20 × LD_50_ BoNT/B, the theoretically neutralization capacity was approximately 40000 × LD_50_/mg, which was the equivalent of 4 IU/mg; 0.28 µg of B12-hFc could provide 50% protection against 20 × LD_50_ BoNT/B, the theoretically neutralization capacity was approximately 71000 × LD_50_/mg, which was the equivalent of 7 IU/mg; 0.2 µg of B5-hFc could provide 50% protection against 20 × LD_50_ BoNT/B, therefore, the theoretical neutralization capacity was approximately 10^5^ × LD_50_/mg, which was the equivalent of 10 IU/mg; and 0.1 μg of B9-hFc provided 50% protection in mice against 20 × LD_50_, indicating that B9-hFc had the strongest activity to neutralize the BoNT/B, and the theoretical neutralization capacity was about 2 × 10^5^ × LD_50_/mg, which was the equivalent to 20 IU/mg.

### 3.7 Prophylactic efficacy of antibodies *in vivo*

Considering special usage scenarios of antibodies, the prophylactic efficacy of the antibodies were investigated. We conducted protective experiments on B5-hFc, B9-hFc, and B12-hFc in a mouse model. The positive and negative controls in the high-dose and low-dose groups comprised BAT-B and T23-hFc, respectively. Different doses of antibodies or PBS were injected into mice via their tail vein. Mice were challenged by intraperitoneal injection with 20 or 100 × LD_50_ BoNT/B at 24, 48, and 72 h post injection of the antibodies. As shown in **Table 4**, the irrelevant antibody T23-hFc and PBS had no preventive effect. After pre-treatment with the high dose of the antibody (0.125 mg/kg), B9-hFc and B12-hFc were effective against 100 × LD_50_ BoNT/B within 3 days, while B5-hFc had protective effect within 1 day. Pretreatment with these three antibodies could completely protect mice exposed to 20 × LD_50_ BoNT/B within 2 days. In the low dose antibody group (0.025 mg/kg), pretreatment with B9-hFc and B12-hFc antibodies was effective against 20 × LD_50_ BoNT/B within 3 days, while B5-hFc was effective within 1 days. Moreover, B9-hFc was effective against 100 × LD_50_ BoNT/B within 3 days. Mice pretreated with the antibodies showed alleviated symptoms or delayed progression to death, demonstrating the significant protective effects of the antibodies. These results suggested that B5-hFc, B9-hFc, and B12-hFc showed prophylactic efficacy against BoNT/B and the protection was dose and pretreatment time-dependent.

**Table 4:** Prophylactic efficacy of the antibodies against BoNT/B in a mouse model.

Antibody^a^	Dose of antibody^b^	Dose of BoNT/B ^c^	Number of survivors/total mice per group		
			24 h^d^	48 h^d^	72 h^d^
B5-hFc	0.025	20 × LD_50_	4/4	0/4	0/4
		100 × LD_50_	1/4	0/4	0/4
	0.125	20 × LD_50_	4/4	4/4	0/4
		100 × LD_50_	1/4	0/4	0/4
B9-hFc	0.025	20 × LD_50_	4/4	4/4	4/4
		100 × LD_50_	4/4	4/4	4/4
	0.125	20 × LD_50_	4/4	4/4	4/4
		100 × LD_50_	4/4	4/4	4/4
B12-hFc	0.025	20 × LD_50_	2/4	1/4	1/4
		100 × LD_50_	1/4	0/4	0/4
	0.125	20 × LD_50_	4/4	4/4	2/4
		100 × LD_50_	2/4	2/4	1/4
BAT-B	0.025	20 × LD_50_	4/4	3/4	3/4
	0.125	100 × LD_50_	4/4	4/4	4/4
T23-hFc	0.025	20 × LD_50_	0/4	0/4	0/4
		100 × LD_50_	0/4	0/4	0/4
	0.125	20 × LD_50_	0/4	0/4	0/4
		100 × LD_50_	0/4	0/4	0/4
PBS	–	20 × LD_50_	0/4	0/4	0/4

a Mice exposed to BoNT were i.v. injected with B5-hFc, B9-hFc or B12-hFc. BAT-B or T23-hFc at the indicated times before exposure. The PBS group was the negative control.

b BoNT/B-exposed mice were injected with 0.025 or 0.125 mg/kg antibody, or PBS at the indicated times before exposure.

c KM mice were i.p. injected with 20 or 100 × LD_50_ of BoNT/B, respectively.

d Mice were i.v. injected with the indicated doses of antibody at 24, 48, or 72 h before exposure to BoNT/B. Data show the number of mice that remained alive (survivors).

### 3.8 Therapeutic efficacy of antibodies *in vivo*

To further investigate the therapeutic efficacy of antibodies, we conducted treatment experiments using B5-hFc, B9-hFc, and B12-hFc, which had the greatest neutralizing activities in the mouse model. Mice were challenged by intraperitoneal injection of 5 or 20 × LD_50_ BoNT/B. Different doses of antibodies or PBS were injected i.v. at 0.5, 1, 2, and 3 h after toxin injection. The controls comprised BAT-B, T23-hFc, and PBS groups. As shown in **Table 5**, the irrelevant antibody T23-hFc and PBS could not protect the mice. In the high dose group (20 × LD_50_ per mouse), 0.125 mg/kg of B5-hFc, B12-hFc, and B9-hFc treatment were effective up to 1, 2 and 3 h after exposure. For the low dose BoNT/B exposure (5 × LD_50_ per mouse), 0.125 mg/kg of B5-hFc, B12-hFc, and B9-hFc treatment was effective up to 3 h after exposure. In addition, 0.025 mg/kg of B5-hFc or B9-hFc treatment was effective up to 3 h after exposure. Treatment in the early stage of mouse botulism, especially before the onset of symptoms, was the best way to protect mice from exposure to BoNT/B. In summary, our results suggested that three antibodies are effective to treat BoNT/B botulism in mice, and the therapeutic effect was dose and pretreatment time dependent.

**Table 5:** Therapeutic efficacy of the antibodies against BoNT/B in a mouse model.

Antibody^a^	Dose of antibody^b^	Dose of BoNT/B^c^	Number of survivors/total mice per group^d^			
			0.5 h^d^	1 h^d^	2 h^d^	3 h^d^
B5-hFc	0.025	5 × LD_50_	4/4	2/4	2/4	2/4
		20 × LD_50_	0/4	0/4	0/4	0/4
	0.125	5 × LD_50_	4/4	4/4	4/4	3/4
		20 × LD_50_	4/4	2/4	0/4	0/4
B9-hFc	0.025	5 × LD_50_	4/4	4/4	4/4	4/4
		20 × LD_50_	4/4	4/4	0/4	0/4
	0.125	5 × LD_50_	4/4	4/4	4/4	4/4
		20 × LD_50_	4/4	4/4	3/4	1/4
B12-hFc	0.025	5 × LD_50_	4/4	4/4	0/4	0/4
		20 × LD_50_	0/4	0/4	0/4	0/4
	0.125	5 × LD_50_	4/4	4/4	3/4	1/4
		20 × LD_50_	4/4	3/4	2/4	0/4
BAT-B	0.025	5 × LD_50_	3/4	2/4	0/4	0/4
	0.125	20 × LD_50_	4/4	4/4	3/4	2/4
T23-hFc	0.025	5 × LD_50_	0/4	0/4	0/4	0/4
		20 × LD_50_	0/4	0/4	0/4	0/4
	0.125	5 × LD_50_	0/4	0/4	0/4	0/4
		20 × LD_50_	0/4	0/4	0/4	0/4
PBS	–	5 × LD_50_	0/4	0/4	0/4	0/4

a Mice exposed to BoNT/B were i.v. injected with B5-hFc, B9-hFc, B12-hFc, BAT-B, or T23-hFc (a neutralizing heavy chain antibody against TeNT), and PBS at the indicated times after exposure.

b BoNT/B-exposed mice were treated with 0.025 or 0.125 mg/kg antibody or PBS at specific times after exposure.

c KM mice were i.p. injected with 5 or 20 × LD_50_ of BoNT/B, respectively.

d Mice were i.v. injected with the indicated doses of antibody at 0.5, 1, 2, or 3 h after exposure to BoNT/B. Data show the number of mice that remained alive (survivors).

## 4 Discussion

In many countries, Botulinum neurotoxins are listed as a category A (highest priority) bioterror agents by the Centers for Disease Control and Prevention because of their extreme potency, lethality, ease of production, and ease of transportation. Prolonged medical care is essential after exposure to these agents (38, 39). Currently, specific treatment is not available for human botulism (40). However, the use of anti-botulinum antibodies in the early stage of poisoning symptoms was reported as the only effective and specific treatment (41). Monoclonal antibodies (mAbs) have been proven to be effective in neutralizing BoNT/B in treatment and were thought to having a lower risk of side effects than antitoxins, such as serum sickness and allergic reactions (10). However, the study of mAbs against BoNT/B is not as extensive as that of BoNT/A. To date, there is no effective neutralizing antibody against BoNT/B for clinical use. NTM-1632, which was an equimolar mixture of three human IgG monoclonal antibodies targeting BoNT/B, is under human clinical trial to assess its safety (14). Therefore, it is of great significance to screen neutralizing antibodies against BoNT/B.

Most of the neutralizing antibodies against botulinum neurotoxin have focused on serotype A (37, 42–45). Numerous reports have described either murine or human BoNT/B-neutralizing antibodies (10, 46–48). In these studies, M2, which specifically binds to the light chain of BoNT/B, showed neutralization activity (approximately 10 × LD_50_/100 µg of the antibody) in a mouse model, and M4, which binds to the BHc, showed partial protection. The combination of M2 (1.25 µg) and M4 (1.25 µg), was able to completely neutralize BoNT/B (80 × LD_50_/2.5 µg of the antibodies) (10). The SC12 antibody showed significant dose-dependent protection against 10 × LD_50_ (39). Only 10 μg of BTBH-N1 completely neutralized 20 × LD_50_ of BoNT/B. However, 100 μg of antibody failed to protect mice challenged with 100 × LD_50_, only prolonging the time to death (48). A previous study reported three neutralizing mAbs that provided complete protection at doses up to 20 × LD_50_ for 100 μg of antibodies (47). So far, there have been relatively few studies on nanobodies against BoNT/B and not effective neutralizing antibodies for clinical use have been reported. Herein, we isolated several nanobodies with potent neutralizing activity: 0.5 μg of B5-hFc, B9-hFc, B12-hFc, B14-hFc, or B20-hFc could completely neutralize 20 × LD_50_ BoNT/B. These antibodies had a much higher neutralizing activity than the antibodies reported in previous studies.

The Hc domain is non-toxic and most of the previously identified neutralizing epitopes were located on the Hc domain (49–51). Previous results from our group also confirmed that immunization with the Hc domain of BoNT/B has a good immunoprotective effect, which can effectively stimulate the body to produce protective antibodies against BoNT/B. Therefore, in this study, BHc was used as antigen to immunize the camel. Herein, we demonstrated that neutralizing nanobodies B5-hFc, B9-hFc, and B12-hFc were effective prophylactically and therapeutically in a mouse model exposed to BoNT/B, and their protective effect is related to the duration and dose of treatment. The results also suggested that the neutralization mechanism of these antibodies functions by blocking the binding of BoNT/B to the receptors on the surface of neuronal cells.

There are several potential limitations of this study. The neutralizing epitopes of BoNT are widely distributed over the whole toxin; thus, the application of single antibody is limited. A combination of antibodies against different domains of BoNT (such as the HN or L domains) could improve the neutralization effect, which is supported by previous studies (10, 14). Another effective strategy is to construct multivalent or multispecific antibodies by enhancing the combination of monoclonal antibodies (37). In the future, we might screen for effective antibodies against other domains of BoNT/B to produce combination or multispecific antibodies. Moreover, the prediction of binding sites of antibody-BoNT/B complexes depends on peptide ELISA and homology modeling applications, and in the future we will use X-ray diffraction or frozen electron microscopy to study the crystal structure parameters.

In conclusion, we constructed a phage display nanobody library from a camel immunized using the purified Hc domain of BoNT/B. Twenty-four specific antibodies against BoNT/B were identified from the library, with different sequences and strong binding. Three heavy chain antibodies, B5-hFc, B9-hFc, and B12-hFc, showed high binding activity for BHc and excellent neutralizing activity against BoNT/B *in vivo*. The three antibodies have potential utility as effective biological agents for prophylactic and therapeutic purposes against BoNT/B *in vivo*.

## Data availability statement

The raw data supporting the conclusions of this article will be made available by the authors, without undue reservation.

## Ethics statement

The animal study was approved by IACUC-DWZX-2022-007 and IACUC-DWZX-2023-037. The study was conducted in accordance with the local legislation and institutional requirements.

## Author contributions

YJ: Data curation, Writing – original draft, Methodology. RW: Data curation, Methodology, Writing – review & editing. JG: Methodology, Writing – review & editing. KC: Methodology, Writing – review & editing. LC: Methodology, Writing – review & editing. XW: Methodology, Writing – review & editing. YL: Methodology, Writing – review & editing. PD: Data curation, Writing – review & editing. CG: Data curation, Writing – review & editing. JL: Writing – review & editing, Writing – original draft, Data curation, Methodology, Project administration. YY: Writing – review & editing, Project administration, Methodology, Writing – original draft. ZY: Writing – review & editing, Project administration, Methodology, Writing – original draft.

## References

[1] Rossetto O Montecucco C. Tables of toxicity of botulinum and tetanus neurotoxins. Toxins (Basel). (2019) 11. doi: 10.3390/toxins11120686 PMC6950492 31771110

[2] Fabris F Sostaric P Matak I Binz T Toffan A Simonato M. Detection of VAMP proteolysis by tetanus and botulinum neurotoxin type B *in vivo* with a cleavage-specific antibody. Int J Mol Sci. (2022) 23. doi: 10.3390/ijms23084355 PMC9024618 35457172

[3] Fan Y Barash JR Lou J Conrad F Marks JD Arnon SS. Immunological characterization and neutralizing ability of monoclonal antibodies directed against botulinum neurotoxin type H. J Infect Dis. (2016) 213:1606–14. doi: 10.1093/infdis/jiv770 PMC4837907 26936913

[4] Maslanka SE Luquez C Dykes JK Tepp WH Pier CL Pellett S. A novel botulinum neurotoxin, previously reported as serotype H, has a hybrid-like structure with regions of similarity to the structures of serotypes A and F and is neutralized with serotype A antitoxin. J Infect Dis. (2016) 213:379–85. doi: 10.1093/infdis/jiv327 PMC4704661 26068781

[5] Yao G Lam KH Perry K Weisemann J Rummel A Jin R. Crystal structure of the receptor-binding domain of botulinum neurotoxin type HA, also known as type FA or H. Toxins (Basel). (2017) 9. doi: 10.3390/toxins9030093 PMC5371848 28282873

[6] Rasetti-Escargueil C Avril A Miethe S Mazuet C Derman Y Selby K. The european antibotABE framework program and its update: Development of innovative botulinum antibodies. Toxins (Basel). (2017) 9. doi: 10.3390/toxins9100309 PMC5666356 28974033

[7] Koepke R Sobel J Arnon SS. Global occurrence of infant botulism, 1976-2006. Pediatrics. (2008) 122:e73–82. doi: 10.1542/peds.2007-1827 18595978

[8] Harris R Tchao C Prystajecky N Cutler J Austin JW. A summary of surveillance, morbidity and microbiology of laboratory-confirmed cases of infant botulism in Canada, 1979-2019. Can Commun Dis Rep. (2021) 47:322–8. doi: 10.14745/ccdr.v47i78a05 PMC8340675 34421389

[9] Dressler D Saberi FA Barbosa ER. Botulinum toxin: mechanisms of action. Arq Neuropsiquiatr. (2005) 63:180–5. doi: 10.1590/s0004-282x2005000100035 15830090

[10] Matsumura T Amatsu S Misaki R Yutani M Du A Kohda T. Fully human monoclonal antibodies effectively neutralizing botulinum neurotoxin serotype B. Toxins (Basel). (2020) 12. doi: 10.3390/toxins12050302 PMC7291131 32392791

[11] Schiavo G Matteoli M Montecucco C. Neurotoxins affecting neuroexocytosis. Physiol Rev. (2000) 80:717–66. doi: 10.1152/physrev.2000.80.2.717 10747206

[12] Montecucco C Rossetto O Schiavo G. Presynaptic receptor arrays for clostridial neurotoxins. Trends Microbiol. (2004) 12:442–6. doi: 10.1016/j.tim.2004.08.002 15381192

[13] Nayak SU Griffiss JM McKenzie R Fuchs EJ Jurao RA An AT. Safety and pharmacokinetics of XOMA 3AB, a novel mixture of three monoclonal antibodies against botulinum toxin A. Antimicrob Agents Chemother. (2014) 58:5047–53. doi: 10.1128/AAC.02830-14 PMC4135817 24913160

[14] Guptill JT Raja SM Juel VC Walter EB Cohen-Wolkowiez M Hill H. Safety, tolerability, and pharmacokinetics of NTM-1632, a novel mixture of three monoclonal antibodies against botulinum toxin B. Antimicrob Agents Chemother. (2021) 65:e0232920. doi: 10.1128/AAC.02329-20 33875433 PMC8218613

[15] Hamers-Casterman C Atarhouch T Muyldermans S Robinson G Hamers C Songa EB. Naturally occurring antibodies devoid of light chains. Nature. (1993) 363:446–8. doi: 10.1038/363446a0 8502296

[16] Kinoshita S Nakakido M Mori C Kuroda D Caaveiro JMM Tsumoto K. Molecular basis for thermal stability and affinity in a VHH: Contribution of the framework region and its influence in the conformation of the CDR3. Protein Sci. (2022) 31:e4450. doi: 10.1002/pro.4450 36153698 PMC9601775

[17] Jovcevska I Muyldermans S. The therapeutic potential of nanobodies. BioDrugs. (2020) 34:11–26. doi: 10.1007/s40259-019-00392-z 31686399 PMC6985073

[18] Hmila I Saerens D Ben Abderrazek R Vincke C Abidi N Benlasfar Z. A bispecific nanobody to provide full protection against lethal scorpion envenoming. FASEB J. (2010) 24:3479–89. doi: 10.1096/fj.09-148213 20410443

[19] Muyldermans S. Nanobodies: natural single-domain antibodies. Annu Rev Biochem. (2013) 82:775–97. doi: 10.1146/annurev-biochem-063011-092449 23495938

[20] De Genst E Silence K Decanniere K Conrath K Loris R Kinne J. Molecular basis for the preferential cleft recognition by dromedary heavy-chain antibodies. Proc Natl Acad Sci U.S.A. (2006) 103:4586–91. doi: 10.1073/pnas.0505379103 PMC1450215 16537393

[21] Wesolowski J Alzogaray V Reyelt J Unger M Juarez K Urrutia M. Single domain antibodies: promising experimental and therapeutic tools in infection and immunity. Med Microbiol Immunol. (2009) 198:157–74. doi: 10.1007/s00430-009-0116-7 PMC2714450 19529959

[22] Muyldermans S Cambillau C Wyns L. Recognition of antigens by single-domain antibody fragments: the superfluous luxury of paired domains. Trends Biochem Sci. (2001) 26:230–5. doi: 10.1016/S0968-0004(01)01790-X 11295555

[23] Klarenbeek A El Mazouari K Desmyter A Blanchetot C Hultberg A de Jonge N. Camelid Ig V genes reveal significant human homology not seen in therapeutic target genes, providing for a powerful therapeutic antibody platform. MAbs. (2015) 7:693–706. doi: 10.1080/19420862.2015.1046648 26018625 PMC4622956

[24] Dong J Thompson AA Fan Y Lou J Conrad F Ho M. A single-domain llama antibody potently inhibits the enzymatic activity of botulinum neurotoxin by binding to the non-catalytic alpha-exosite binding region. J Mol Biol. (2010) 397:1106–18. doi: 10.1016/j.jmb.2010.01.070 PMC2903050 20138889

[25] Tremblay JM Vazquez-Cintron E Lam KH Mukherjee J Bedenice D Ondeck CA. Camelid VHH antibodies that neutralize botulinum neurotoxin serotype E intoxication or protease function. Toxins (Basel). (2020) 12. doi: 10.3390/toxins12100611 PMC7598594 32987745

[26] Lam KH Perry K Shoemaker CB Jin R. Two VHH antibodies neutralize botulinum neurotoxin E1 by blocking its membrane translocation in host cells. Toxins (Basel). (2020) 12. doi: 10.3390/toxins12100616 PMC7599855 32992561

[27] Baghban R Gargari SL Rajabibazl M Nazarian S Bakherad H. Camelid-derived heavy-chain nanobody against Clostridium botulinum neurotoxin E in Pichia pastoris. Biotechnol Appl Biochem. (2016) 63:200–5. doi: 10.1002/bab.1226 24673401

[28] Fu Y da Fonseca Rezende EMJ Fleming BD Renn A Chen CZ Hu X. A humanized nanobody phage display library yields potent binders of SARS CoV-2 spike. PLoS One. (2022) 17:e0272364. doi: 10.1371/journal.pone.0272364 35947606 PMC9365158

[29] Ye G Gallant JP Massey C Shi K Tai W Zheng J. The development of a novel nanobody therapeutic for SARS-CoV-2. bioRxiv. (2020). doi: 10.1101/2020.11.17.386532

[30] de Smit H Ackerschott B Tierney R Stickings P Harmsen MM. A novel single-domain antibody multimer that potently neutralizes tetanus neurotoxin. Vaccine X. (2021) 8:100099. doi: 10.1016/j.jvacx.2021.100099 34169269 PMC8207222

[31] Shi DY Liu FJ Li ZY Mao YY Lu JS Wang R. Development and evaluation of a tetravalent botulinum vaccine. Hum Vaccin Immunother. (2022) 18:2048621. doi: 10.1080/21645515.2022.2048621 35435814 PMC9196761

[32] Chen L Lu J Yue J Wang R Du P Yu Y. A humanized anti-human adenovirus 55 monoclonal antibody with good neutralization ability. Front Immunol. (2023) 14:1132822. doi: 10.3389/fimmu.2023.1132822 37006289 PMC10060833

[33] Liu FJ Shi DY Li ZY Lu JS Wang R Pang XB. Evaluation of a recombinant tetanus toxin subunit vaccine. TOXICON. (2020) 187:75–81. doi: 10.1016/j.toxicon.2020.08.001 32889026

[34] Shi DY Chen BY Mao YY Zhou G Lu JS Yu YZ. Development and evaluation of candidate subunit vaccine against botulinum neurotoxin serotype B. Hum Vaccin Immunother. (2019) 15:755–60. doi: 10.1080/21645515.2018.1547613 PMC6988880 30433836

[35] Shi DY Lu JS Mao YY Liu FJ Wang R Du P. Characterization of a novel tetravalent botulism antitoxin based on receptor-binding domain of BoNTs. Appl Microbiol Biotechnol. (2023) 107:3205–16. doi: 10.1007/s00253-023-12515-2 PMC10102682 37058230

[36] Froude JW Stiles B Pelat T Thullier P. Antibodies for biodefense. MAbs. (2011) 3:517–27. doi: 10.4161/mabs.3.6.17621 PMC3242838 22123065

[37] Lu J Jiang Y Guo J Chen L Liu F Li Z. A human bispecific antibody neutralizes botulinum neurotoxin serotype A. Sci Rep. (2023) 13:20806. doi: 10.1038/s41598-023-48008-5 38012220 PMC10681988

[38] Hicks RP Hartell MG Nichols DA Bhattacharjee AK van Hamont JE Skillman DR. The medicinal chemistry of botulinum, ricin and anthrax toxins. Curr Med Chem. (2005) 12:667–90. doi: 10.2174/0929867053202223 15790305

[39] Wang H Li T Shi J Cai K Hou X Wang Q. A new neutralizing antibody against botulinum neurotoxin B recognizes the protein receptor binding sites for synaptotagmins II. Microbes Infect. (2010) 12:1012–8. doi: 10.1016/j.micinf.2010.07.002 20650331

[40] Thanongsaksrikul J Chaicumpa W. Botulinum neurotoxins and botulism: a novel therapeutic approach. Toxins (Basel). (2011) 3:469–88. doi: 10.3390/toxins3050469 PMC3202833 22069720

[41] Sobel J. Botulism. Clin Infect Dis. (2005) 41:1167–73. doi: 10.1086/444507 16163636

[42] Adekar SP Jones RM Elias MD Al-Saleem FH Root MJ Simpson LL. A human monoclonal antibody that binds serotype A botulinum neurotoxin. Hybridoma (Larchmt). (2008) 27:11–7. doi: 10.1089/hyb.2007.0536 18294071

[43] Mowry MC Meagher M Smith L Marks J Subramanian A. Production and purification of a chimeric monoclonal antibody against botulinum neurotoxin serotype A. Protein Expr Purif. (2004) 37:399–408. doi: 10.1016/j.pep.2004.06.022 15358363

[44] Yu R Wang S Yu YZ Du WS Yang F Yu WY. Neutralizing antibodies of botulinum neurotoxin serotype A screened from a fully synthetic human antibody phage display library. J Biomol Screen. (2009) 14:991–8. doi: 10.1177/1087057109343206 19726786

[45] Xiong X Qiu Y Zheng J Zhou L Wang Q Pang J. Generation and characterization of a monoclonal antibody against FGFR3 that protects mice from BoNT/A. Protein Expr Purif. (2024) 213:106370. doi: 10.1016/j.pep.2023.106370 37709211

[46] Fan Y Dong J Lou J Wen W Conrad F Geren IN. Monoclonal antibodies that inhibit the proteolytic activity of botulinum neurotoxin serotype/B. Toxins (Basel). (2015) 7:3405–23. doi: 10.3390/toxins7093405 PMC4591640 26343720

[47] Chen C Wang S Wang H Mao X Zhang T Ji G. Potent neutralization of botulinum neurotoxin/B by synergistic action of antibodies recognizing protein and ganglioside receptor binding domain. PLoS One. (2012) 7:e43845. doi: 10.1371/journal.pone.0043845 22952786 PMC3430616

[48] Yang GH Kim KS Kim HW Jeong ST Huh GH Kim JC. Isolation and characterization of a neutralizing antibody specific to internalization domain of Clostridium botulinum neurotoxin type B. Toxicon. (2004) 44:19–25. doi: 10.1016/j.toxicon.2004.03.016 15225558

[49] Hanson MA Stevens RC. Cocrystal structure of synaptobrevin-II bound to botulinum neurotoxin type B at 2.0 A resolution. Nat Struct Biol. (2000) 7:687–92. doi: 10.1038/77997 10932255

[50] Dertzbaugh MT West MW. Mapping of protective and cross-reactive domains of the type A neurotoxin of Clostridium botulinum. Vaccine. (1996) 14:1538–44. doi: 10.1016/s0264-410x(96)00094-1 9014296

[51] Atassi MZ Dolimbek BZ Hayakari M Middlebrook JL Whitney B Oshima M. Mapping of the antibody-binding regions on botulinum neurotoxin H-chain domain 855-1296 with antitoxin antibodies from three host species. J Protein Chem. (1996) 15:691–700. doi: 10.1007/BF01886751 8968960

