# Supplementary material for: Isolation and characterization of Hc-targeting chimeric heavy chain antibodies neutralizing botulinum neurotoxin type B
Source: Front Immunol. 2024 Apr 30;15:1380694. doi: 10.3389/fimmu.2024.1380694 (PMC11109933; doi:10.3389/fimmu.2024.1380694)
Supplement: Supplementary file 1 [file DataSheet_1.pdf]

frontiers

Supplementary Material

Isolation and characterization of Hc-targeting chimeric heavy chain antibodies neutralizing botulinum neurotoxin type B

Yujia Jiang, Rong Wang, Jiazheng Guo, Kexuan Cheng, Lei Chen, Xi Wang, Yating Li, Peng Du, Chen Gao, Jiansheng Lu*, Yunzhou Yu*, Zhixin Yang*

*** Correspondence:** Jiansheng Lu, lujiansheng2008@163.com

Yunzhou Yu, yunzhouyu@163.com

Zhixin Yang, yy_xiao@126.com

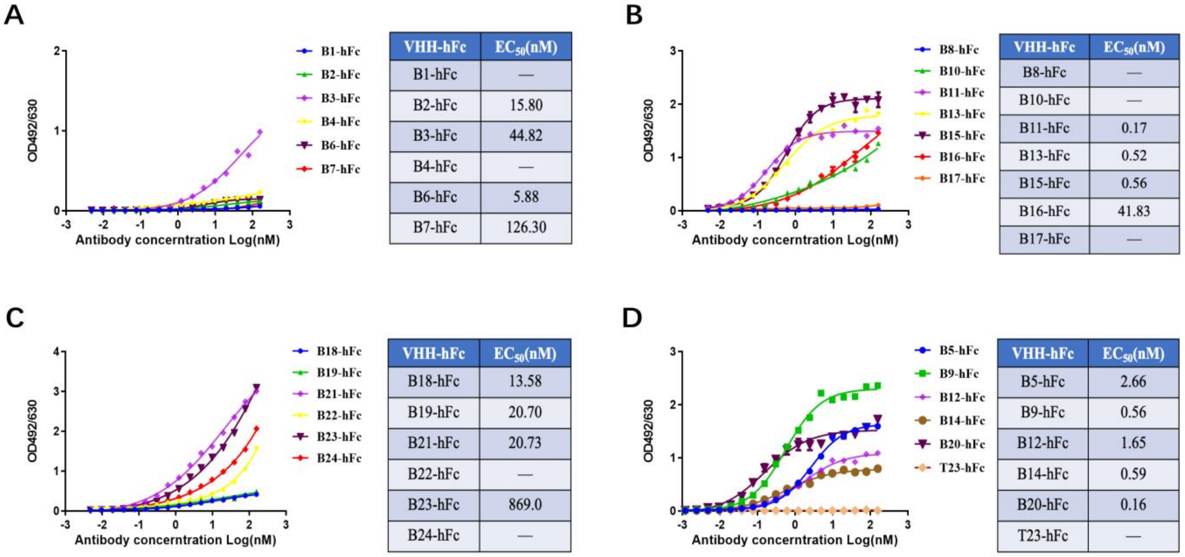

Supplementary Figure 1. Binding activity profiles of antibodies towards BHc. (A) Binding profiles for B1-hFc, B2-hFc, B3-hFc, B4-hFc, B6-hFc and B7-hFc toward BHc. (B) Binding profiles for B8-hFc, B10-hFc, B11-hFc, B13-hFc, B15-hFc, B16-hFc and B17-hFc toward BHc. (C) Binding profiles for B18-hFc, B19-hFc, B20-hFc, B21-hFc, B22-hFc, B23-hFc and B24-hFc toward BHc. (D) Binding profiles for B5-hFc, B9-hFc, B12-hFc, B14-hFc, B20-hFc and T23-hFc toward BHc. The initial concentration of all antibodies was 12.5 μg/mL. The antibodies were serially diluted

with each subsequent concentration halved. The plates were coated with BHc at 200 ng per well to assess binding activity.

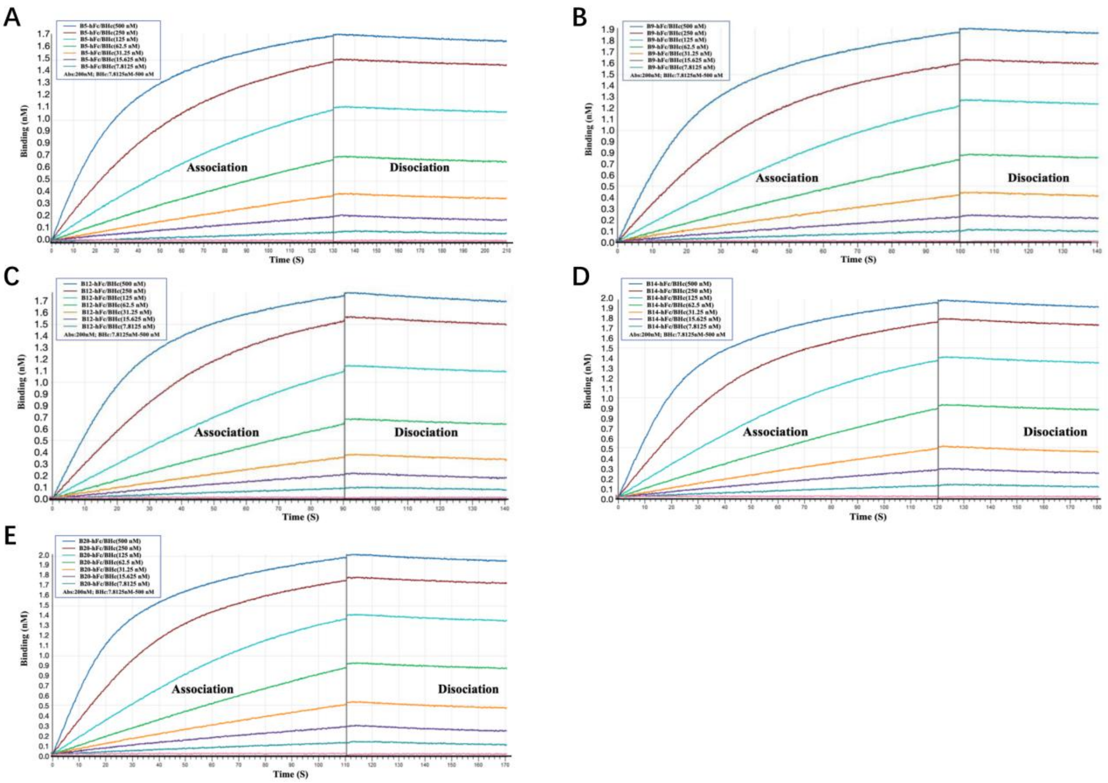

Supplementary Figure 2. The kinetics processes of the five antibodies binding to BHc detected by BLI. The antibodies included are B5-hFc(A), B9-hFc(B), B12-hFc(C), B14-hFc(D) and B20-hFc(E). Antibodies at a concentration of 200 nM were immobilized on the biosensor for determination. BHc was two-fold diluted from 500 nM to 7.8125 nM. The samples were separated by slash "/", separately indicating "Association" and "Disassociation" stages of sample molecules.
